# Attenuation of irradiated choroid and its regional vortex veins in central serous chorioretinopathy after photodynamic therapy

**DOI:** 10.1038/s41598-023-47325-z

**Published:** 2023-11-14

**Authors:** Hidetaka Matsumoto, Junki Hoshino, Kosuke Nakamura, Shoji Kishi, Hideo Akiyama

**Affiliations:** Department of Ophthalmology, Gunma University Graduate School of Medicine, 3-39-15 Showa-Machi, Maebashi, Gunma 371-8511 Japan

**Keywords:** Diseases, Eye diseases, Macular degeneration

## Abstract

We retrospectively studied 12 eyes of 12 patients with central serous chorioretinopathy (CSC) to investigate choroidal thickness changes following half-fluence photodynamic therapy (PDT) using widefield choroidal thickness maps obtained by optical coherence tomography (OCT). Additionally, we assessed the relationship between choroidal thickness changes and the regional vortex veins as visualized on widefield en face OCT of the choroid. Pre-treatment en face images of the choroidal vasculature were superimposed on subtracted choroidal thickness maps before and 3 months after half-fluence PDT. The choroidal thickness decreased mainly in the irradiated macular area and in the region of vortex veins which function as drainage for the macula in all eyes. Eleven eyes (91.7%) showed choroidal thinning in the nasal area which overlapped with the nasal vortex vein distribution. Moreover, in 10 (90.9%) of those eyes, we observed intervortex venous anastomosis across the vertical watershed zone. Quantitative analysis revealed that the reduction in choroidal thickness was most pronounced in the macular area. Furthermore, the choroidal thickness reduction in the area with macular drainage vortex veins was significantly greater than that in the area without such vortex veins. These results suggest that half-fluence PDT might decrease choroidal thickness due to choriocapillaris occlusion in the irradiated macula, possibly leading to diminished venous drainage from the macula to regional vortex veins. Moreover, venous blood flow through the anastomotic vessels from the macular drainage vortex veins into the nasal vortex veins might be reduced post-treatment.

## Introduction

The term “pachychoroid” refers to choroidal thickening which is associated with dilated outer choroidal vessels^1^. Various pachychoroid spectrum diseases, including central serous chorioretinopathy (CSC), have been described^2,3^. Previous studies identified several characteristic findings of pachychoroid spectrum diseases, including asymmetric dilatation of vortex veins^4^, intervortex venous anastomosis^5,6^, geographic filling delay of the choriocapillaris^7,8^, pulsatile vortex venous flow^9,10^, and choroidal vascular hyperpermeability^11^. These findings suggest vortex venous stasis to possibly play a pathophysiological role in pachychoroid spectrum diseases^12,13^. Notably, most of these features have been replicated in animal models of vortex venous congestion developed by our group^14,15^.

Common treatment approaches for CSC include focal laser photocoagulation targeting the leakage point(s), as identified by fluorescein angiography (FA), and photodynamic therapy (PDT) targeting the area of choroidal vascular hyperpermeability (CVH), as detected by indocyanine green angiography (ICGA). Prior studies employing optical coherence tomography (OCT) have demonstrated significantly reduced choroidal thickness following PDT^16^. On the other hand, focal laser photocoagulation often does not produce a marked decrease in choroidal thickness^16,17^. Moreover, post-PDT changes include reduced dilatation of outer choroidal vessels, as seen on OCT, and decreased CVH, as demonstrated by ICGA^18,19^. Furthermore, investigations utilizing laser speckle flowgraphy revealed diminished choroidal blood flow after PDT administered for CSC^20^. These findings, taken together, suggest the potential effectiveness of PDT for ameliorating the vortex venous stasis that is associated with CSC.

In recent years, the advent of widefield swept-source OCT has enabled the assessment of choroidal thickness not only in the macular but also the peripheral area. Studies employing widefield choroidal thickness maps have shown that CSC eyes exhibit choroidal thickening in both macular and peripheral areas, as compared to normal eyes^21,22^. It has also been reported that after PDT for CSC, choroidal thickness decreases not only in the macular but also in the peripheral area^23,24^. However, little research has focused on the potential relationship between choroidal thickness changes after PDT for CSC and the functions of vortex veins. Herein, we investigated the choroidal thickness changes following PDT for CSC using widefield choroidal thickness maps. We also assessed the relationship between the observed changes and the vortex vein distribution as visualized in widefield en face OCT images of the choroid.

## Results

The baseline demographic and clinical characteristics of our CSC patients treated with half-fluence PDT are listed in Table 1. The subjects analyzed were all Japanese. We studied 12 eyes of 12 patients in total [8 men (66.7%) and 4 women (33.3%), average age: 59.2 ± 11.8 years] with treatment-naïve CSC. The average of the greatest linear dimension (GLD) value was 2179 ± 1126 μm. Macular drainage vortex veins, i.e., dilated vortex veins functioning as drainage routes for the macula, were superotemporal in 4 eyes (33.3%) and inferotemporal in 2 (16.7%), while 6 eyes (50.0%) had combined superotemporal/inferotemporal macular drainage vortex veins.

**Table 1:** Baseline demographic and clinical characteristics of CSC patients treated with half-fluence photodynamic therapy.

Number of eyes	12	
Number of patients	12	
Age (years)	59.2 ± 11.8	
Male	8 (66.7%)	
Best-corrected visual acuity (logMAR)	0.19 ± 0.26	
Macular drainage vortex veins	Superotemporal	4 (33.3%)
	Inferotemporal	2 (16.7%)
	Combined	6 (50.0%)
Central choroidal thickness (µm)	435 ± 86	
Greatest linear dimension (µm)	2179 ± 1126	

**Figure 1:**
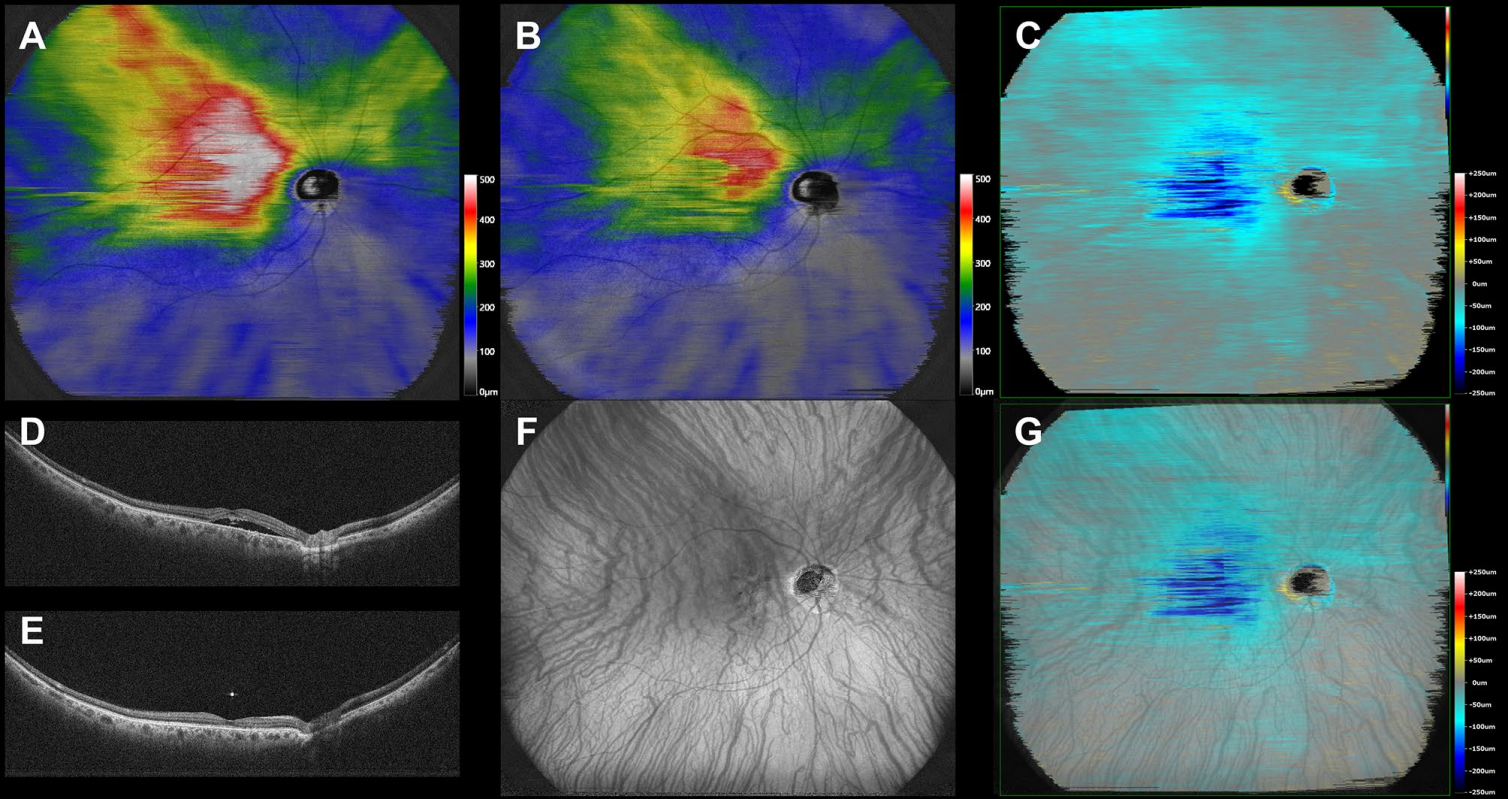
Images are from the right eye of a 70-year-old man with central serous chorioretinopathy with superotemporal macular drainage vortex veins. (**A**) Widefield choroidal thickness map shows thickened choroid at the posterior pole area as well as in the superotemporal and superonasal areas. (**B**) Three months after half-fluence photodynamic therapy (PDT), widefield choroidal thickness map shows a reduction in choroidal thickening. (**C**) Subtracted wide-field choroidal thickness map shows the area of diminished choroidal thickness. (**D**) Horizontal B-mode optical coherence tomography (OCT) through the fovea shows choroidal thickening associated with dilated outer choroidal vessels accompanied by subretinal fluid. The central choroidal thickness is 432 µm. (**E**) Three months after half-fluence PDT, horizontal B-mode OCT through the fovea shows diminished choroidal thickening with no subretinal fluid. The central choroidal thickness is 292 µm. (**F**) Pre-treatment widefield en face OCT image of the choroid shows superotemporal macular drainage vortex veins. Anastomoses can be seen between the superotemporal and superonasal vortex veins superior to the papilla. (**G**) Superimposed image of the pre-treatment widefield en face OCT and the subtracted widefield choroidal thickness map reveals that choroidal thickness decreased mainly in the macular area and the region corresponding to the superotemporal macular drainage vortex veins. The nasal area of diminished choroidal thickness overlaps the superonasal vortex vein distribution.

**Figure 2:**
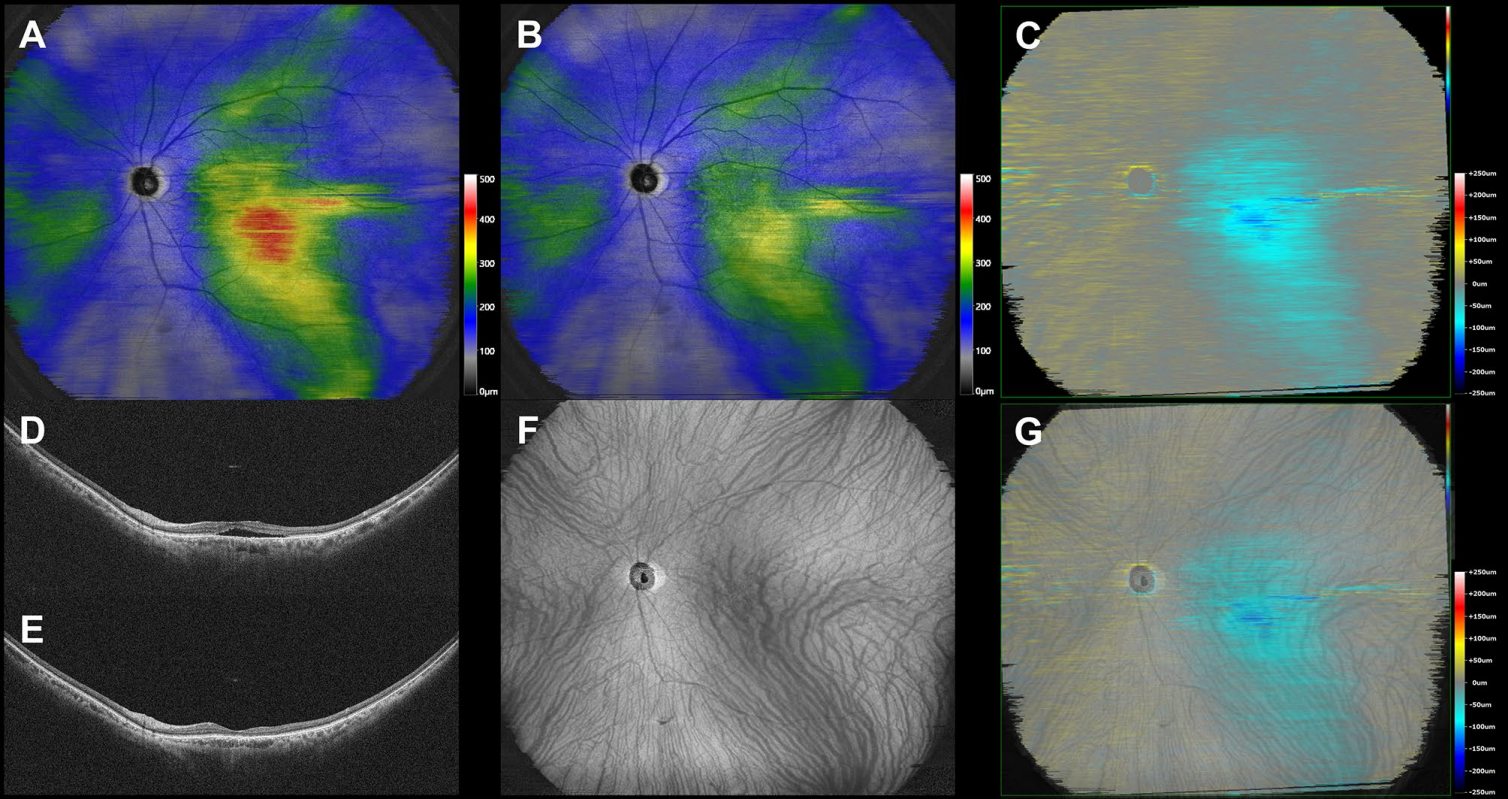
Images are from the left eye of a 66-year-old man with central serous chorioretinopathy with inferotemporal macular drainage vortex veins. (**A**) Widefield choroidal thickness map shows thickened choroid at the posterior pole area and in the inferotemporal areas. (**B**) Three months after half-fluence photodynamic therapy (PDT), widefield choroidal thickness map shows a reduction in choroidal thickening. (**C**) Subtracted widefield choroidal thickness map shows the area of diminished choroidal thickness. (**D**) Horizontal B-mode optical coherence tomography (OCT) through the fovea shows choroidal thickening associated with dilated outer choroidal vessels accompanied by subretinal fluid. The central choroidal thickness is 316 µm. (**E**) Three months after half-fluence PDT, horizontal B-mode OCT through the fovea shows reduced choroidal thickening with no subretinal fluid. The central choroidal thickness is 221 µm. (**F**) Pre-treatment widefield en face OCT image of the choroid shows inferotemporal macular drainage vortex veins. There are no anastomoses between the inferotemporal and nasal vortex veins. (**G**) Superimposed image of the pre-treatment widefield en face OCT and the subtracted widefield choroidal thickness map reveals choroidal thickness to have decreased mainly in the macular area and the region corresponding to the inferotemporal macular drainage vortex veins.

**Figure 3:**
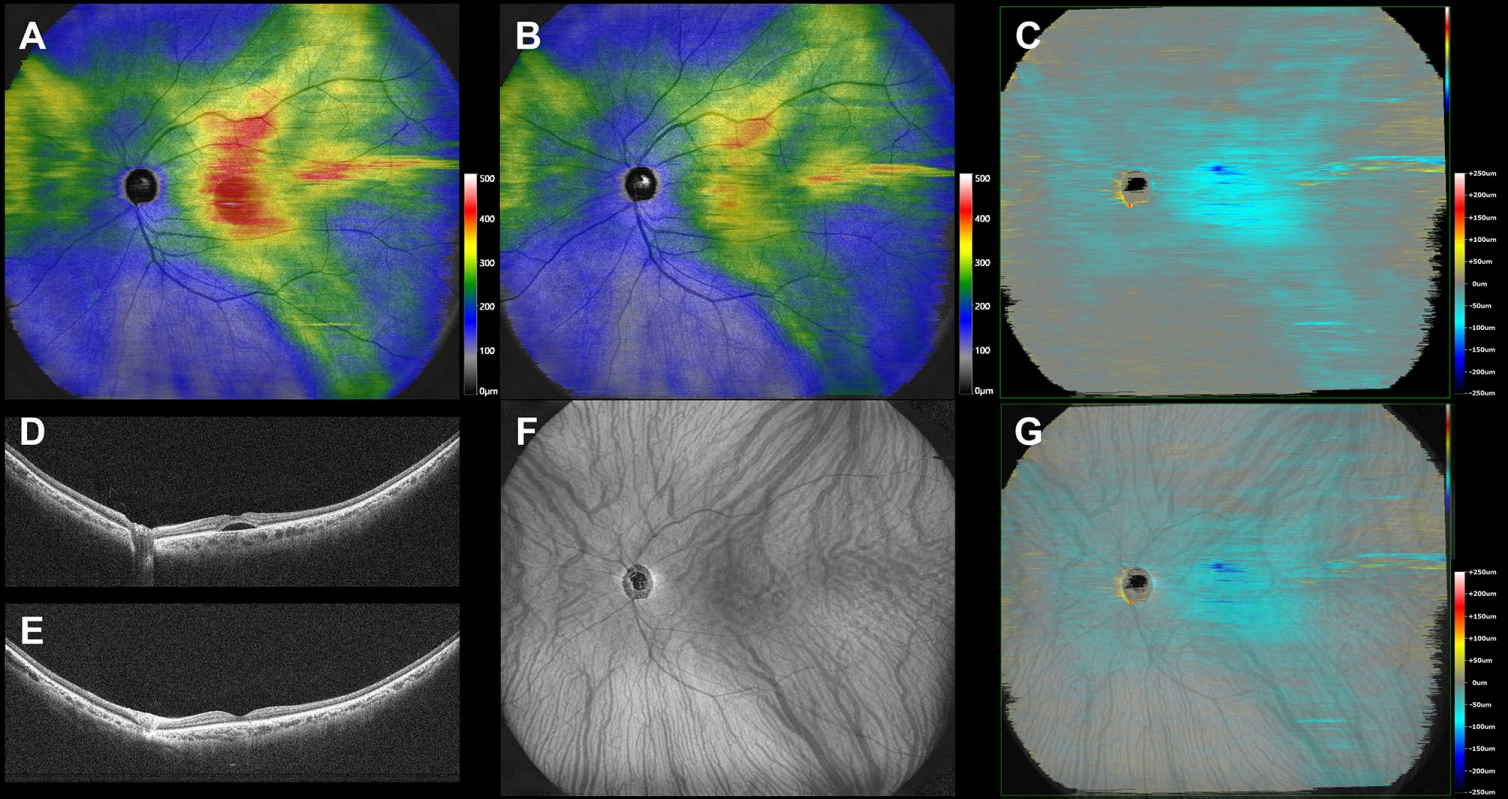
Images from the left eye of a 61-year-old woman with central serous chorioretinopathy with superotemporal and inferotemporal macular drainage vortex veins. (**A**) Widefield choroidal thickness map shows thickened choroid at the posterior pole area as well as in the superotemporal, inferotemporal, and superonasal areas. (**B**) Three months after half-fluence photodynamic therapy (PDT), widefield choroidal thickness map shows a reduction in choroidal thickening. (**C**) Subtracted widefield choroidal thickness map shows the area of diminished choroidal thickness. (**D**) Horizontal B-mode optical coherence tomography (OCT) through the fovea shows choroidal thickening associated with dilated outer choroidal vessels accompanied by subretinal fluid. The central choroidal thickness is 412 µm. (**E**) Three months after half-fluence PDT, horizontal B-mode OCT through the fovea shows reduced choroidal thickening with no subretinal fluid. The central choroidal thickness is 336 µm. (**F**) Pre-treatment widefield en face OCT image of the choroid shows superotemporal and inferotemporal macular drainage vortex veins. Anastomoses can be seen between the superotemporal and superonasal vortex veins superior to the papilla. (**G**) Superimposed image of the pre-treatment widefield en face OCT and subtracted widefield choroidal thickness map reveals choroidal thickness to have decreased mainly in the macular area and the region corresponding to the superotemporal and inferotemporal macular drainage vortex veins. The nasal area of reduced choroidal thickness overlaps the superonasal vortex vein distribution.

Superimposed images of the choroidal vasculature in pre-treatment widefield en face OCT images and subtracted widefield choroidal thickness maps before and 3 months after half-fluence PDT revealed choroidal thickness to have decreased mainly in the irradiated macular area and the region corresponding to the macular drainage vortex veins in all 12 eyes. Eleven eyes (91.7%) also showed choroidal thinning in the nasal area which overlapped with the nasal vortex vein distribution. Moreover, 10 (90.9%) of these 11 eyes had intervortex venous anastomosis across the vertical watershed zone. Representative cases are presented in Figs. 1, 2, and 3.

**Figure 4:**
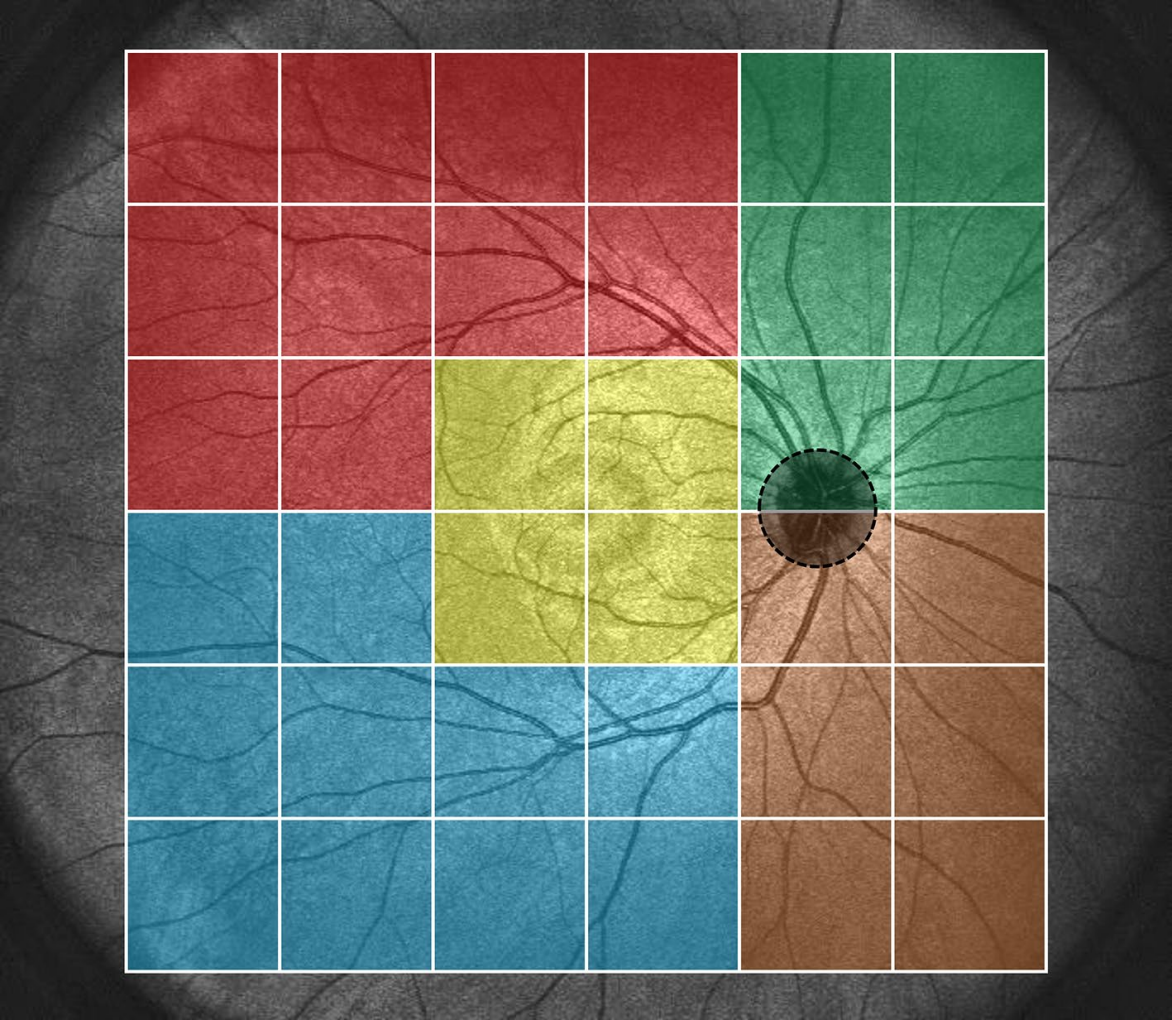
Quantitative assessment of choroidal thickness employing OCT Research Tool. A 6 × 6 square grid sector of 3 × 3 mm was applied to the widefield choroidal thickness maps centered on the fovea. The mean choroidal thickness within each square grid sector was automatically computed. The 18 × 18 mm area was divided into 5 sections: the macular area consisting of 4 squares (yellow), superotemporal with 10 squares (red), inferotemporal with 10 squares (blue), superonasal with 6 squares (green), and inferonasal with 6 squares (brown). This division of areas outside the macular area was based on presumed horizontal and vertical watershed zones. The mean choroidal thickness in each of these areas was calculated. A 2.5 mm-in-diameter circular area centered on the papilla was excluded from the measurements.

**Figure 5:**
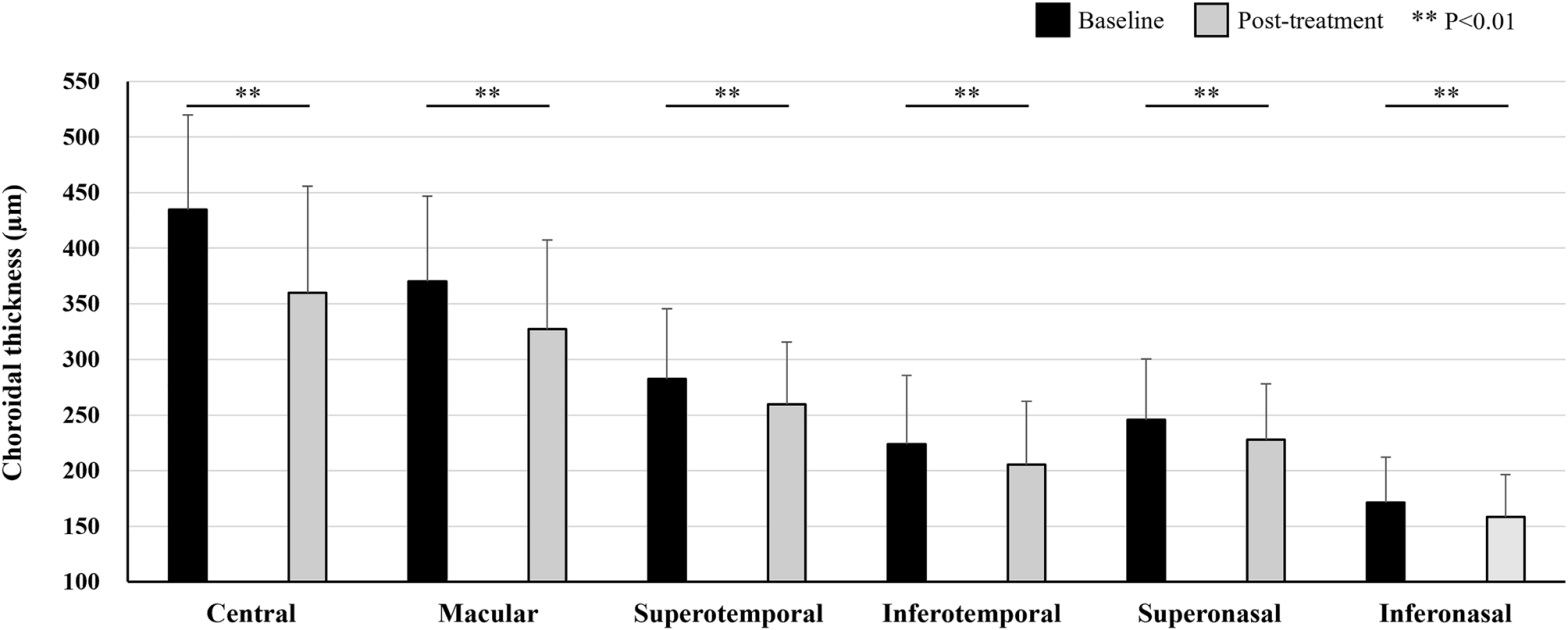
Choroidal thicknesses before and 3 months after half-fluence photodynamic therapy for central serous chorioretinopathy. Choroidal thickness significantly decreased in each area of the fundus after treatment.

**Figure 6:**
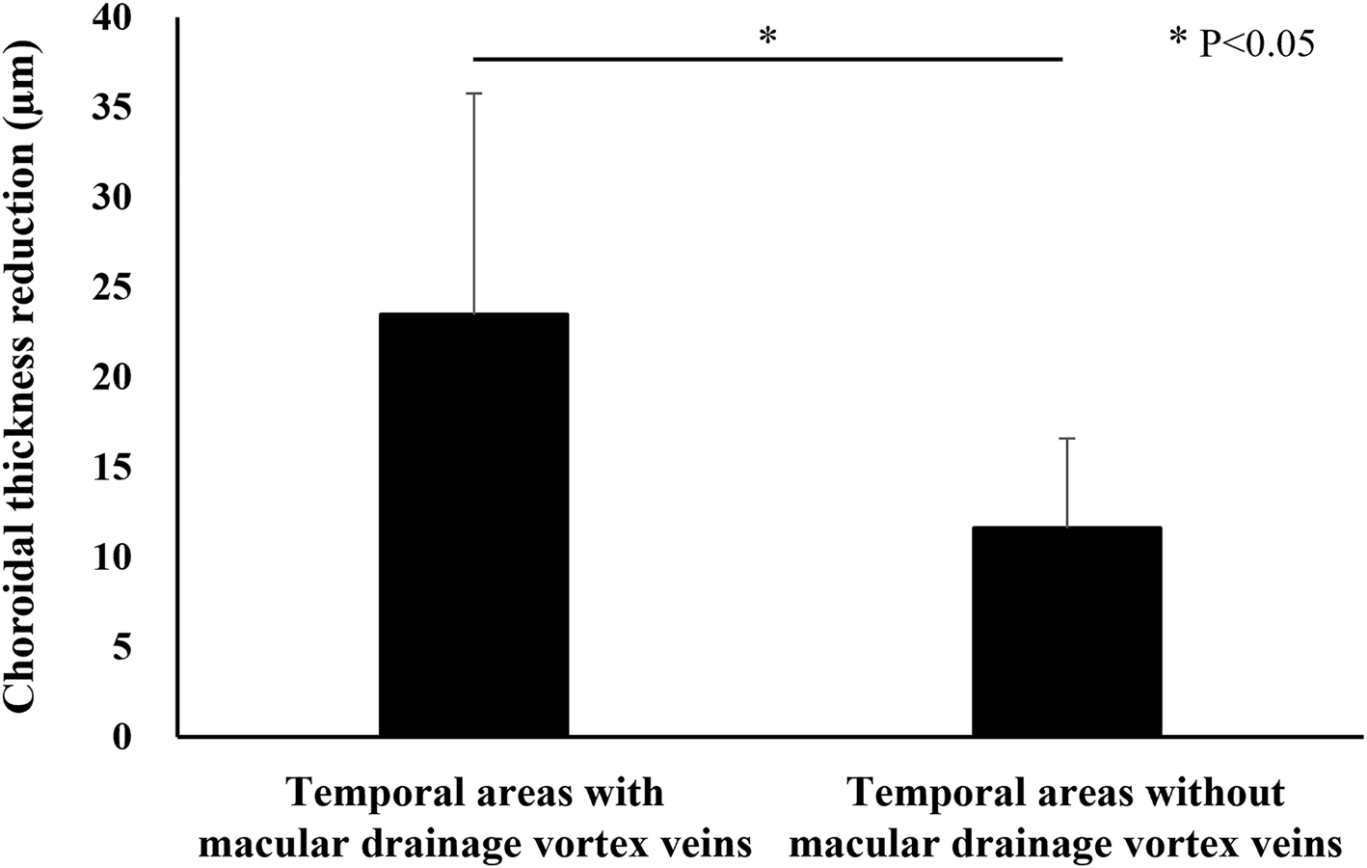
Choroidal thickness reductions in superotemporal and inferotemporal areas with or without macular drainage vortex veins 3 months after half-fluence photodynamic therapy for central serous chorioretinopathy. Reductions in choroidal thickness in the superotemporal and inferotemporal areas with macular drainage vortex veins were significantly greater than those in the superotemporal or inferotemporal areas without such vortex veins.

Central choroidal thickness (CCT) was 435 ± 86 µm at baseline, showing a significant reduction to 360 ± 96 µm (P < 0.01) at 3 months after half-fluence PDT. Additionally, the mean choroidal thicknesses in the macular, superotemporal, inferotemporal, superonasal, and inferonasal areas were 370 ± 77, 282 ± 63, 224 ± 62, 246 ± 55, and 171 ± 41 µm, respectively, at baseline. These thicknesses significantly decreased to 327 ± 80, 260 ± 56, 205 ± 57, 228 ± 50, and 158 ± 38 µm, respectively, 3 months post-treatment (all P < 0.01) (Figs. 4, 5). The choroidal thickness reductions were 43 ± 15, 23 ± 14, 18 ± 9, 18 ± 12, and 13 ± 7 µm, respectively, with the most notable decrease being observed in the macular area. Moreover, the choroidal thickness reductions in the superotemporal and inferotemporal areas with macular drainage vortex veins were significantly greater than those in the superotemporal or inferotemporal areas without such vortex veins (23 ± 12 vs. 12 ± 5 µm, P < 0.05) (Fig. 6).

Three months post-treatment, serous retinal detachment (SRD) had been fully absorbed in 10 eyes (83.3%) and was diminished in the other 2 (16.7%). Best-corrected visual acuity (BCVA) was 0.19 ± 0.26 at baseline, showing an improvement to 0.05 ± 0.22 (P < 0.01) at 3 months after the treatment.

## Discussion

We employed widefield choroidal thickness maps obtained by OCT to determine choroidal thickness changes in eyes with CSC after half-fluence PDT, and then investigated the possible relationship between changes in choroidal thickness and the vortex vein distribution as visualized on widefield en face OCT. Diminished choroidal thickness was noted mainly in the irradiated macular area and the region corresponding to the superotemporal and/or inferotemporal macular drainage vortex veins in all of the eyes studied. The choroidal thickness in the nasal area was also reduced in most of our cases, and this overlapped with the nasal vortex vein region. We frequently observed intervortex venous anastomosis across the vertical watershed zone.

Prior investigations employing widefield choroidal thickness maps detected alterations in choroidal thickness in eyes with CSC after PDT^23,24^. In both earlier investigations, the 100-degree or 18 mm-in-diameter circular field centered on the fovea was subdivided into 9 areas. These studies documented a significant post-PDT reduction in the average choroidal thickness across all areas. Consistent with the results of these previous studies, our research revealed a significant choroidal thickness reduction in all 5 areas within the 18 × 18 mm square area centered on the fovea 3 months after half-fluence PDT. Notably, the reduction in choroidal thickness was most pronounced in the macular area. Moreover, the choroidal thickness reductions in the superotemporal and inferotemporal areas with macular drainage vortex veins were significantly greater than those in the superotemporal or inferotemporal area without such vortex veins. These findings support the idea that choroidal thickness decreased mainly in the macular area and in the regions corresponding to the macular drainage vortex veins.

The mechanisms underlying choroidal thickness reduction not only in the macular but also the peripheral area after PDT for CSC have not as yet been fully elucidated. Another study examined human eye tissue extracted one week after PDT for choroidal melanoma using electron microscopy and found destruction of vascular endothelial cells of the choriocapillaris within the treated area, leading to vascular occlusion^25^. Based on our present observations, we speculate that partial occlusion of the choriocapillaris in the irradiated area diminished blood inflow from the short posterior ciliary arteries and subsequently decreased blood drainage from the macula into the regional vortex veins. Therefore, the peripheral choroidal thickness reduction might be substantial in the region corresponding to the vortex veins draining the macula.

Funatsu et al. suggested the possible existence of intervortex venous anastomosis across the vertical watershed zone in CSC eyes based on thinning of the choroid from the macula to the nasal area after PDT^23^. In our present study, nasal-side choroidal thickness after half-fluence PDT was reduced in 11 out of 12 eyes (91.7%), with vortex vein anastomosis across the vertical watershed zone being noted in 10 of these eyes (90.9%). These results indicate that venous blood flow through the anastomotic vessels from the macular drainage vortex veins into the nasal vortex veins might have been reduced post-treatment. In our monkey model of vortex vein congestion, temporal vortex veins anastomosed with the nasal vortex veins after ligation of the superotemporal and inferotemporal vortex veins outside the sclera^15^. This phenomenon suggests that there can be compensation for blood congestion in the temporal vortex veins by blood drainage from the temporal to the nasal vortex veins via anastomotic vessels. Similarly, in CSC eyes, anastomotic vessels may have arisen between temporal and nasal vortex veins as a compensatory response to vortex venous stasis. Therefore, choroidal thickness reduction developed not only in the region of macular drainage vortex veins but also in that of nasal vortex veins in response to half-fluence PDT.

Limitations of this study include its single-center, retrospective design, small sample size, short-term follow-up, having only Japanese participants, and relying primarily on qualitative assessments. However, to our knowledge, this is the first report to demonstrate the relationship between choroidal thickness changes after half-fluence PDT and the vortex vein distribution using widefield choroidal thickness maps and widefield en face OCT images with automatic segmentation of the choroid.

In conclusion, after half-fluence PDT for CSC, due to partial choriocapillaris occlusion, blood inflow from the short posterior ciliary arteries to the choriocapillaris might be reduced, subsequently leading to a decrease in venous blood draining into the macular drainage vortex veins. This in turn may lead to diminished choroidal thickness, mainly in the irradiated macular area and the region corresponding to the macular drainage vortex veins.

## Methods

Approval was obtained from the Institutional Review Board of Gunma University Hospital and we adhered to the guidelines of the Declaration of Helsinki in carrying out this study. Informed consent was obtained from all individual participants included in the study. We retrospectively studied 12 eyes of 12 patients with previously untreated CSC accompanied by persistent SRD for more than 3 months. Patients received half-fluence PDT during the period from January 2022 through April 2023. All 12 were followed for more than 3 months at Gunma University Hospital.

Before starting the treatment regimen, all 12 patients underwent a complete baseline ophthalmological examination including BCVA, intraocular pressure, slit-lamp biomicroscopy with a noncontact fundus lens (SuperField lens; Volk Optical Inc., Mentor, OH), color fundus photography (Canon CX-1; Canon, Tokyo, Japan, and California; Optos, Dunfermline, Scotland, UK), swept-source OCT (Xephilio OCT-S1; Canon Inc., Tokyo, Japan, and PLEX Elite 9000; Carl Zeiss Meditec, Dublin, CA, USA), FA and ICGA (Spectralis HRA + OCT; Heidelberg Engineering, Heidelberg, Germany, and California; Optos). We assessed retinochoroidal structures employing Xephilio OCT-S1 with 1010–1110 nm near infrared illumination and a 100,000 A-scans/second scanning speed. We obtained 3-dimensional volume data of vertical 20 mm (1024 B-scans) × horizontal 23 mm (1024 pixels) scans, centered on the fovea, using enhanced depth imaging of swept source OCT. OCT angiography (OCTA) volume scanning, i.e., 300 × 300 pixels in the 3 × 3 mm area including FA leakage point(s), was performed employing the PLEX Elite 9000 with a 1050 nm central wavelength and a 100,000 A-scans/second scanning speed. The OCTA was based on optical microangiography algorithm. FA and ICGA with an angle of 30 degrees centered on the fovea were obtained utilizing the Spectralis HRA + OCT.

CSC was diagnosed if all of the following criteria were met. (1) SRD was confirmed by color fundus photography and on OCT images. (2) FA showed dye leakage within the SRD. (3) Macular neovascularization was ruled out based on FA, ICGA, and OCTA images. CSC patients were excluded from this study if even one of the following exclusion criteria was met. (1) Presence of any other diseases involving the fundus, (2) Any history of prior treatment for CSC, (3) Any history of being prescribed steroid medication, (4) CCT of less than 300 µm or more than 600 µm in the eye affected by CSC. The last criterion was applied because it is difficult to detect choroidal thickness changes in eyes with CCT of less than 300 µm as well as to identify the chorioscleral interface in eyes with CCT exceeding 600 µm.

All eyes underwent half-fluence PDT. Patients were given a verteporfin injection at 6 mg/m^2^ body surface area and then received PDT, for 83 s, with a light fluence of 25 J/cm^2^ using a Visulas PDT system 690S (Carl Zeiss Japan, Tokyo, Japan). GLD was determined to cover the CVH area detected by ICGA, including leakage point(s) seen on FA. The PDT treatment spot size diameter was the GLD plus 1000 µm.

Three-dimensional volume data were again acquired 3 months after half-fluence PDT utilizing the Xephilio OCT-S1. The total choroid was automatically segmented using built-in software supported by artificial intelligence, and we set the choroidal thickness as the vertical distance from Bruch's membrane to the chorioscleral interface. Whenever the automatic segmentation had been incorrectly performed, we manually corrected all of the segmentation errors. We generated a subtracted widefield choroidal thickness map from the widefield choroidal thickness maps obtained before and 3 months post-treatment employing an OCT Research Tool Ver. 2.0. Moreover, we examined widefield en face OCT images of the choroid and identified macular drainage vortex veins that drain the venous blood from the macular area. The macular drainage vortex veins were then classified into 3 groups: superotemporal, inferotemporal, and combined superotemporal/inferotemporal vortex veins. We superimposed the subtracted choroidal thickness map onto the pre-treatment en face OCT image of the choroid for each eye, which allowed qualitative assessment of the relationship between choroidal thickness changes and the vortex vein distribution. Moreover, we conducted a quantitative analysis of the widefield choroidal thickness maps obtained using the OCT Research Tool Ver. 2.0. As shown in Fig. 4, a 6 × 6 square grid sector of 3 × 3 mm was applied to the widefield choroidal thickness maps centered on the fovea. The mean choroidal thickness within each square grid sector was automatically computed. Subsequently, we divided the 18 × 18 mm area into 5 sections: macular, superotemporal, inferotemporal, superonasal, and inferonasal. This division of areas outside the macular area was based on presumed horizontal and vertical watershed zones. The mean choroidal thickness in each of these areas was calculated and compared before and 3 months post-treatment. A 2.5 mm-in-diameter circular area centered on the papilla was excluded from the measurements.

For statistical analysis, the Wilcoxon signed-rank test was employed to compare the differences in choroidal thickness and BCVA before and after treatment. Unpaired values for choroidal thickness were compared using the Mann–Whitney U test. Data analyses were performed with Excel (Microsoft, Redmond, WA, USA) using the add-in software Statcel4^26^. A value of P < 0.05 was taken to indicate a statistically significant difference. All data are presented as average ± standard deviation.

## Data Availability

The datasets used and/or analyzed during the current study are available from the corresponding author upon reasonable request.
